# Cognitive plausibility in voice-based AI health counselors

**DOI:** 10.1038/s41746-020-0278-7

**Published:** 2020-05-15

**Authors:** Thomas Kannampallil, Joshua M. Smyth, Steve Jones, Philip R. O. Payne, Jun Ma

**Affiliations:** 1 Department of Anesthesiology, Washington University School of Medicine, St Louis, MO USA; 2 Institute for Informatics, Washington University School of Medicine, St Louis, MO USA; 3 Department of Biobehavioral Health, The Pennsylvania State University, University Park, PA USA; 4 Department of Communication, University of Illinois at Chicago, Chicago, IL USA; 5 Department of Medicine, University of Illinois at Chicago, Chicago, IL USA

**Keywords:** Health services, Translational research

## Abstract

Voice-based personal assistants using artificial intelligence (AI) have been widely adopted and used in home-based settings. Their success has created considerable interest for its use in healthcare applications; one area of prolific growth in AI is that of voice-based virtual counselors for mental health and well-being. However, in spite of its promise, building realistic virtual counselors to achieve higher-order maturity levels beyond task-based interactions presents considerable conceptual and pragmatic challenges. We describe one such conceptual challenge—cognitive plausibility, defined as the ability of virtual counselors to emulate the human cognitive system by simulating how a skill or function is accomplished. An important cognitive plausibility consideration for voice-based agents is its ability to engage in meaningful and seamless interactive communication. Drawing on a broad interdisciplinary research literature and based on our experiences with developing two voice-based (voice-only) prototypes that are in the early phases of testing, we articulate two conceptual considerations for their design and use—conceptualizing voice-based virtual counselors as communicative agents and establishing virtual co-presence. We discuss why these conceptual considerations are important and how it can lead to the development of voice-based counselors for real-world use.

## Introduction

Artificial intelligence (AI) affords new opportunities for the practice of medicine ranging from early disease detection to precision medicine tools for cancer treatment^1,2^. The promise of AI is accompanied with corresponding hype regarding its potential for “revolutionizing the healthcare for patients and populations”^3^. One area of prolific growth in AI, especially in home-based settings, has been voice-based (or voice-only) personal assistants (e.g., Amazon’s Alexa). Recent reports have suggested that the worldwide sales of Amazon’s Alexa and Google Home have crossed 100 million and 50 million, respectively (https://bit.ly/3b5AkKS). However, their use has primarily focused on routine tasks including playing music, initiating conversations (e.g., “Alexa, Good morning”), searching for information (e.g., weather), and performing smart home functions based on voice instructions^4^. Their high adoption notwithstanding, use of voice-based AI technologies may be declining over time^5^, attributable to the lack of advanced features for seamless voice interactions^6^. User evaluation studies of voice-based assistants have characterized the conversational interactions similar to “having a bad personal assistant”^7^.

Nonetheless, recent initiatives have catalyzed the use of such technologies in the healthcare domain. For example, the United Kingdom’s National Health Services wants to enable Alexa devices for patients to find answers to routine questions such as “what are the symptoms of chicken pox?” (https://www.bbc.com/news/health-48925345). Despite considerable skepticism regarding their pragmatic potential for reliable patient interactions^8^, there have been some efforts to integrate voice-based applications within the patient care continuum. One domain where voice assistants are expected to grow is that of serving as health coaches or therapists (hereafter referred to as counselors).

Voice-based virtual counselors—as opposed to early forms of automated counseling that involve static interactions, often with text messages or menu-based interfaces—allow for relatively unscripted interactions^9^. These interactions, with seemingly natural communication, can be sensitive to patients’ cognitive and emotional states, providing contextually relevant and empirically supported counseling, and thereby, promoting a sense of connection with their “virtual counselor.” Such virtual counselors afford opportunities for behavioral health and wellness promotion, given the shortage of clinicians, reimbursement and many barriers to patient access to mental and preventive health services.

These voice-based counselors are different from a variety of prior innovations for virtual counseling. For example, embodied conversational agents (ECA)^10,11^ utilized both voice-based and direct manipulation interfaces (e.g., user selection from a list) for clinical and cognitive support in various domains, including marriage counseling^12^, palliative care counseling^13^, and collecting family health history^14^. A more direct comparisons for the voice-based virtual counselors are the text-based “chat bots” that provided text-based emotional support^9^. A comprehensive review of these existing (predominantly, non-voice based) virtual counseling applications is beyond the scope of this article. Here we focus on the need to develop a fundamental conceptualization that is aligned with the cognitive processes underlying human communicative processes in order to realize the potential of a voice-based virtual counselor for intelligent, “human-like” conversations. Examples of similar cognitive alignment have been shown to affect a number of aspects of healthcare tools, such as usability, workflow, and patient safety^15^.

For virtual counselors, given their focus on primarily voice-based interactions, a significant step would be the conceptualization of its *cognitive plausibility*. Cognitive plausibility is the ability of a system to emulate the human cognitive system by simulating how a skill or function is accomplished^16^. In more simplistic terms, cognitive plausibility is characterized as the functional performance of a system where the inputs and outputs to a system are comparable to that of humans^17^ (a more well-known equivalent example is the “Turing test”).

For a voice-based virtual counselor, cognitive plausibility refers to its capability of engaging in and replicating conversations that mirror patient conversations with a human counselor. This can include the ability to identify patients’ reasons of current concern, their medical or psychological problems, relate to or reference their past counseling sessions, assess their current emotional state(s) and contextual situations, develop a shared understanding regarding problems, and create a plausible action plan to address them with follow-up resolutions or further treatment options for unresolved problems.

Achieving cognitive plausibility in voice-based virtual counselors is challenging due to an array of technical and socio-technical constraints. In this comment, we describe two pragmatic considerations for developing cognitively plausible voice-based agents: creating them as communicative agents and establishing co-presence during communicative interactions. To exemplify the applicability of these conceptual considerations, we discuss them within the context of the design and development of two virtual counselor prototypes for mental health and emotional well-being on Amazon’s Alexa platform—a counselor delivering problem-solving therapy for managing depression and anxiety and a counselor for mindfulness-based stress therapy.

## Virtual counselors as communicative agents

Developing cognitive plausibility in voice-based virtual counselors requires a change regarding our conceptualization of AI-based conversational agents: as opposed to considering these as interactive objects for technological task-based transactions, these need to be designed as “communicative subjects”^6^. Within this conceptualization, AI voice assistants are interlocutors and play the dual role facilitating conversations and mediating social processes around interactive communication: social communicative processes such as conversational flexibility (e.g., switching topics), context awareness (e.g., knowing previous conversations), or intent recognition (e.g., inferring implicit intentions). Similarly, the conceptualization of voice-based virtual counselors should embody the role of social actors, and interactions should be anchored around how human counselors would normally channel and orient their interactions with patients undergoing counseling for physical or mental health problems^18,19^. In other words, a primary design and functional consideration for cognitive plausibility should be to design these counselors as communicators, as people will perceive and interact with them in the applicable healthcare context.

One of the ways cognitive plausibility can be achieved is through the management of interactivity in conversations. Human interactive communication is a “joint activity in which two or more interlocutors share or synchronize aspects of their private mental states and act together in the world”^20,21^. Achieving interactivity during conversations is largely dependent on utilizing appropriate conceptual frameworks for managing and supporting interactivity. Three cognitive theories are often used to describe interactive communication and its characteristics: the message model, the two-stage model, and the collaborative or grounding model^22^. The message model of communication is an “information transfer” framework based on information theory, where communication involves the transmission of informational content from a sender to a receiver through a channel^23^. In this framework, there is no understanding of nuances or intent of communication, with the focus merely being on transmitting and interpreting the informational content. In contrast, the two-stage model of communication relies on interactive alignment between conversational partners, where the content of communication of one partner primes the other, leading to a shared mental representation regarding the presented content^24,25^. Conversational continuity relies on priming and convergent communicative behaviors, which are typically achieved through mimicry and behavioral adjustments by interpreting and aligning (often, unconsciously) with a partner. The third model, representing in our view a relatively more sophisticated explanation of interactive human communication, postulated by Brennan and Clark^26^, describes communication as a joint, collaborative activity. Communicative content and signals are recognized by partners and meanings are coordinated through a process of grounding, where partners provide supporting evidence that they “understand each other” through verbal or situational cues^27^.

These models span the spectrum of human-like interactive communication—with the messaging model representing a mechanistic view of communication, and the two-stage and grounding models representing the nuanced, interpretive, and cognitive principles of interactive communication. This spectrum also broadly exemplifies the degree of complexity in simulating realistic voice-based interactions with a virtual counselor. Most current voice-based applications rely on the messaging model, where specific terminology (e.g., keywords) is used as the basis for routine interactions, without accounting for semantic, structural, temporal, or cognitive aspects of the spoken language. For example, a question “Alexa, what is the weather?” or “Alexa, who is the weather?” would elicit similar responses pertaining to the current local weather (relying on the keyword, “weather”). At the other end of the spectrum, the grounding model is a highly collaborative model that relies on visual and verbal cues from a conversational partner. Replicating such a conversational model in a virtual counselor is currently beyond the scope of voice assistants due to the limitations of technology to characterize human intent, such as identifying non-voice (e.g., gestural, facial) or other situational cues and assessing contextual factors in an environment. Such an interaction requires voice-based virtual counselors to be “stateful,” by aligning voice-based interactions within the context of past/historical interactions.

However, it is potentially plausible for AI-based tools to more readily align with the two-stage messaging model that relies on behavioral adjustments and mimicry through a process of cognitive priming to a partner’s communicative interactions. AI technology, in its current state, is able to detect dynamic changes during voice interactions related to conversational turns (e.g., break and switch between conversations), engage in repair of conversations (e.g., after a conversation breakdown/stoppage)^28–31^, create contextual awareness (e.g., through integration with other sensors), and identify mood states (e.g., happy, sad). Such detection is a preliminary precursor for providing cues for adjustments throughout the conversation.

Our prototype design of the two voice-based virtual counselors was aligned with, and informed by, the two-stage model, incorporating features for interactivity and context-sensitive adaptiveness. First, we created conversational continuity through realistic turns for each speaker (virtual counselor, patient) with timed breaks in conversations. This involved the creation of shortened conversational turns for each speaker; for example, avoiding one participant speaking for an extended period. Virtual counselors speaking turn lengths were kept purposefully short; timed interruptions were incorporated to minimize patient conversational turn length. Shorter mean turn lengths are intended to increase interactivity, reducing the potential for distractions/diversions, and engaging patients in a conversation. Second, we developed considerable adaptivity during conversations, including acknowledgement of patient responses as a proxy for virtual counselor engagement, flexibility in the conversations (e.g., repair from breakdowns rather than abrupt stoppage), and awareness of previous sessions to drive current conversations. These design considerations are aligned with the two-stage model. Both prototypes are in the early phase of user testing, and the findings will inform whether and how the designs help create realistic interactions with voice-based virtual counselors.

## Establishing co-presence

A key characteristic of patient–counselor interaction is their synchronous co-presence during a counseling session (e.g., face-to-face, by phone, or via telehealth means). Such synchronous conversational partners, where individuals are “available, accessible, and subject to one another”^32^ can potentially help to foster a degree of interactivity and facilitates the building of trust and believability between the conversational partners. Such synchronous interactions are referred to as co-presence or social presence^33^. In the case of voice-based virtual counselors, co-presence of the human is with a digital communication agent, and such interactions are referred to as virtual telecopresence^34^.

Aligning interaction designs focusing on establishing virtual telecopresence to enhance the role of virtual counselors as communication agents can potentially help in achieving cognitive plausibility in such interactions. This can be achieved in several ways: first, humans perceive interactive digital devices as social actors^6,35,36^; as such, trust and believability in the virtual counselor can help the patient engage in counseling sessions and adhere to their care plan. Second, creating embodiment, a degree of engagement that is created by the presence of humans in a conversation, can enhance the quality and nature of communicative interactions^13,14^. For example, embodied interactions in the presence of other humans provide cues such as facial expressions, eye contact, and gestures that mediate and drive the conversational interactions. In contrast, voice-only interactions with a virtual counselor have a reduced level of embodiment requiring additional external sources to enhance engagement.

In one of our prototypes, we used a smartwatch for enabling contextually aware responses to create a sense of social presence of the counselor. The purpose of creating such external sensors was as an “add on” for developing more engaging conversations. In on-going early testing, the smartwatch is used to detect stress through ecological momentary assessments; additional activities such as sleep patterns and general physical activities are also captured to create a more situated understanding regarding patient activities. Another promising direction, especially in the case of mental health virtual counselors, are the emotion-sensing features that can determine a patient’s mood states via sentiment analysis and analysis of the acoustic features of speech such as pitch, tone, volume, and timbre. Preliminary evaluations suggest that integration of such sensing in a virtual counselor may help to better situate a conversation, providing not only a feeling of co-presence but also helps in developing rapport and trust in the counselor, potentially strengthening such relationships^37–39^.

Co-presence can also be achieved through persistence and continuity in the interactions. Design features in virtual counselors such as the ability to continue conversations in different settings and situations through their integration in mobile phone or tablets as opposed to being on a tethered device (such as an Amazon device) can help in developing continuity.

## Conclusions

The widespread adoption of voice-based AI assistants has opened new opportunities for its potential role in the delivery of evidence-based health counseling interventions. Based on our experience with developing voice-based virtual counselors for mental health and emotional well-being, we describe the conceptualization of cognitive plausibility in designing such virtual counselors in order to create seamless and seemingly human-like interactive communication. We specifically focus on voice-based virtual counselors relying on AI, as this is a burgeoning area of technology development with little empirical research. In addition, we are at stage where scholarly discourse about conceptual advances can have a meaningful impact on the emerging science. The concept of cognitive plausibility is relevant to the design of any system (e.g., ECAs) that aims to interact with humans in an intelligent, “human-like” manner. However, it is unclear, based on our review, how cognitive plausibility has been accounted in the design of ECAs. We posit that the considerations of conceptual plausibility are necessary to advance a maturity framework for the design of AI-based voice counselors such that they may achieve higher-order maturity levels beyond technological task-based transactions in order to be pragmatically useful and efficacious. Findings from empirical testing of such virtual counselors will help refine and extend these conceptual considerations, contributing to the maturation of this emerging field.
